# Draft Genome Sequence of *Cupriavidus* sp. Strain IK-TO18, Isolated from Antimony-Contaminated Sediment

**DOI:** 10.1128/MRA.00724-21

**Published:** 2021-09-23

**Authors:** Tomotaka Okubo, Nobuyoshi Nakajima, Shigeki Yamamura, Natsuko Hamamura

**Affiliations:** a Graduate School of Systems Life Sciences, Kyushu University, Fukuoka, Fukuoka, Japan; b Biodiversity Division, National Institute for Environmental Studies, Tsukuba, Ibaraki, Japan; c Regional Environment Conservation Division, National Institute for Environmental Studies, Tsukuba, Ibaraki, Japan; d Department of Biology, Faculty of Science, Kyushu University, Fukuoka, Fukuoka, Japan; Indiana University, Bloomington

## Abstract

*Cupriavidus* sp. strain IK-TO18 was isolated from antimony-contaminated sediment. The draft genome sequence of the isolate contains 6,605 predicted protein-coding sequences, including genes associated with heavy metal resistance and the aerobic degradation of aromatic hydrocarbons. This sequence will provide valuable information regarding the functional versatility of the genus *Cupriavidus*.

## ANNOUNCEMENT

Species in the genus *Cupriavidus* are well characterized for their ability to tolerate numerous heavy metals and degrade aromatic compounds (1–3). The versatile metabolisms of *Cupriavidus* species have gained interest as model organisms in bioremediation and geochemical studies. Here, we present the draft genome sequence of *Cupriavidus* sp. strain IK-TO18, which was isolated from antimony-contaminated sediment.

Enrichment culturing was conducted in anaerobically prepared basal salts medium (4) amended with lactate (2 mM) and anthraquinone-2,6-disulfonate (AQDS; 1 mM) and inoculated with antimony-contaminated sediment (containing 12.0 mg kg^−1^ Sb) collected from a former mine site (Ehime, Japan). The cultures were incubated at 25°C, and positive AQDS reduction was visually identified by the production of orange anthrahydroquinone-2,6-disulfonate (AHQDS) (5). Serially diluted positive enrichments were inoculated onto Luria broth plates and incubated aerobically. The colonies were restreaked for isolation, and the obtained pure culture was identified as a Gram-negative *Cupriavidus* sp. by sequencing the 16S rRNA gene and designated strain IK-TO18.

Strain IK-TO18 was grown in basal salts medium with lactate under anaerobic conditions, as described above. Genomic DNA was extracted using the MoBio PowerSoil DNA isolation kit (Qiagen) and fragmented into an average size of 350 bp using an M220 sonicator (Covaris Inc., MA). A paired-end library (insert size, ∼350 bp) was prepared using the NEBNext Ultra DNA library prep kit for Illumina (New England BioLabs). Genome sequencing was performed on the HiSeq X sequencing platform (Illumina, San Diego, CA) at the National Institute for Environmental Studies. A total of 11,668,203 raw paired-end reads were generated (2 × 150 bp), and low-quality sequences (Q ≤ 13) were removed using the Trim_Reads tool implemented in CLC Genomic Workbench 20.0.2 (GW; Qiagen). The sequences were assembled by *de novo* assembly in slow mode in GW with default parameters, except for the minimum contig length (500) and word size (30). The resulting 109 contigs had an *N*_50_ value of 230,767 bp and a maximum contig length of 531,585 bp. The draft genome sequence of strain IK-TO18 was 7,423,042 bp long, with 231.0× genome coverage and a G+C content of 66.1%. Annotation was conducted using the NCBI Prokaryotic Genome Annotation Pipeline (6), resulting in 6,605 predicted protein-coding sequences, 63 tRNAs, and 3 complete rRNAs (1 copy each of 5S, 16S, and 23S). BLASTn analysis of the 16S rRNA gene showed that this strain is closely related to other *Cupriavidus* strains (sequence identity, >98%), such as the copper-resistant bacterium Cupriavidus necator strain N-1 (GenBank accession number NR_102851.1) (3).

The genome annotation identified a variety of genes responsible for heavy metal resistance, including the *ars* operon (arsenic resistance), *mer* operon (mercury resistance), cobalt/zinc/cadmium resistance protein, copper resistance proteins, and ATPases for transport of lead/cadmium, zinc, and mercury. In addition, genes were found encoding enzymes involved in the aerobic degradation of aromatic hydrocarbons, including phenol degradation (phenol hydroxylase), catechol degradation via the *meta-* and *ortho-*cleavage pathways (catechol 2,3-dioxygenase and catechol 1,2-dioxygenase, respectively), and benzoate degradation (benzoate 1,2-dioxyganase). The draft genome sequence of *Cupriavidus* sp. strain IK-TO18 provides valuable information regarding the functional versatility of the genus *Cupriavidus*.

### Data availability.

The draft genome sequence was deposited in GenBank under accession number JADGMP000000000, BioProject accession number PRJNA622630, BioSample accession number SAMN16491722, and SRA accession number SRX9374696.
